# Supplementary material for: Alleviating the burden of diabetes with Health Equity Funds: Economic evaluation of the health and financial risk protection benefits in Cambodia
Source: PLoS One. 2021 Nov 5;16(11):e0259628. doi: 10.1371/journal.pone.0259628 (PMC8570764; doi:10.1371/journal.pone.0259628)
Supplement: S1 Appendix — (DOCX) [file pone.0259628.s001.docx]

# Appendix

## Diabetes-related service packages under the Health Equity Funds

## *Service Package 11: Diabetes screening at health centers*

**Clinical Indications:** This specific service package covers all services involved with screening and counseling persons for and about diabetes of persons with one or more risk factors (frequent urinating during the night, polydipsia, aged *≥*40 years, overweight, tuberculosis, sudden weight loss, melioidosis, hypertension, history of stroke or ischemic heart disease).

#### Services covered:

- - 1. Consultation
       - History taking
       - Vital information like blood pressure, height, weight, waist circumference
       - Fasting blood glucose
    2. Medication
       - Referral to higher-level hospital if necessary
    3. Counseling
       - If present in the operational district: local Peer Educator Group
       - Blood sugar and blood pressure targets bodyweight
       - Behavioral changes regarding diet, exercise, smoking, alcohol
       - Risk factors for diabetic complications and means to prevent these
    4. Non-Medical Benefits
       - Self-management booklet with health education for record keeping
       - A3-Diabetes Pyramid Poster

Source: Guidelines for the Benefit Package and Provider Payment of the Health Equity Fund for the Poor

## *Service Package 12: Diabetes treatment and follow-up at health centers*

**Clinical Indications:** This service package covers the treatment and follow-up of people diagnosed with diabetes mellitus. It is intended to be used for quarterly follow-up consultations to monitor clinical progress once stable. If more or less frequent consultations are needed, use Service Package 11.

#### Services Covered:

Consultation

- - - - History since last consultation, including self-management, symptoms, treatment adherence and side effects, diet, exercise, smoking, alcohol
      - Any glucose of HbA1c results since last consultation
      - Examination for blood pressure, height, weight, waist circumference
    1. Investigations
       - Fasting blood glucose
    2. Medication
       - Medication as indicated by the Clinical Practice Guidelines
       - Insulin syringes
    3. Counseling
       - Encourage diabetes patient to have contact with other diabetes patients in the village and peers
       - Behavioral changes regarding diet, exercise, smoking, alcohol
       - Risk factors for diabetic complications and means to prevent these
       - Adherence with and understanding of requirements for treatment
       - Check ability of self-monitoring, ask to see the patient self-management book and discuss the records and progress towards targets

**Required Documentation:** Letter from referral hospital to continue for treatment and follow up

Source: Guidelines for the Benefit Package and Provider Payment of the Health Equity Fund for the Poor

## *Service Package 30: Diabetes screening at referral hospitals*

**Clinical Indications:** This specific service package covers all services involved with screening and counseling persons for and about diabetes of persons with one or more risk factors (frequent urinating during the night, polydipsia, aged *≥*40 years, overweight, tuberculosis, sudden weight

loss, melioidosis, hypertension, history of stroke or ischemic heart disease).

#### Services covered:

- - 1. Consultation
       - History taking
       - Vital information like blood pressure, height, weight, waist circumference
       - Fasting blood glucose
       - HbA1c, creatin + urine albumin/protein + lipid profile + at least K^+^ electrolyte
    2. Medication
       - Medication as indicated by the Clinical Practice Guidelines
       - Insulin syringes
    3. Counseling
       - If present in the operational district: local Peer Educator Group
       - Blood sugar and blood pressure targets bodyweight
       - Behavioral changes regarding diet, exercise, smoking, alcohol
       - Risk factors for diabetic complications and means to prevent these
    4. Non-Medical Benefits
       - Self-management booklet with health education for record keeping
       - A3-Diabetes Pyramid Poster

#### Required Documentation:

- Standard Patient Dossier Medical Records
- Hospital HEF Summary of Treatment Form
- Referral letter to higher-level hospital if necessary

Source: Guidelines for the Benefit Package and Provider Payment of the Health Equity Fund for the Poor

## *Service Package 31: Diabetes treatment and follow-up at referral hospitals*

**Clinical Indications:** This service package covers the treatment and follow-up of people diagnosed with diabetes mellitus. It is intended to be used for quarterly follow-up consultations to monitor clinical progress once stable. If more or less frequent consultations are needed, use Service Package 32.

#### Services Covered:

- - 1. Consultation
       - History since last consultation, including self-management, symptoms, treatment adherence and side effects, diet, exercise, smoking, alcohol
       - Any glucose of HbA1c results since last consultation
       - Examination for blood pressure, height, weight, waist circumference

Investigations

- - - - Fasting blood glucose or RBG (+/- HbA1c one time per year), urine protein, creatin, lipids, hepatitis C
    1. Medication
       - Medication as indicated by the Clinical Practice Guidelines
       - Insulin syringes
    2. Counseling
       - Encourage diabetes patient to have contact with other diabetes patients in the village and peers
       - Behavioral changes regarding diet, exercise, smoking, alcohol
       - Risk factors for diabetic complications and means to prevent these
       - Adherence with and understanding of requirements for treatment
       - Check ability of self-monitoring, ask to see the patient self-management book, and discuss the records and progress towards targets

#### Required Documentation:

- Standard Patient Dossier Medical Records
- Hospital HEF Summary of Treatment Form
- Referral letter to higher-level hospital if necessary

Source: Guidelines for the Benefit Package and Provider Payment of the Health Equity Fund for the Poor

## *Service Package 32: Uncontrolled diabetes consultation at referral hospitals*

**Clinical Indications:** This service package is to be used for diabetes-related consultations that are not covered by Service Package 31.

#### Services Covered:

- - 1. Consultation
       - History since last consultation, including self-management, symptoms, treatment adherence and side effects, diet, exercise, smoking, alcohol
       - Any glucose of HbA1c results since last consultation
       - Examination for blood pressure, height, weight, waist circumference

Investigations

- - - - Fasting blood glucose or RBG (+/- HbA1c one time per year), urine protein, creatin, lipids, hepatitis C
    1. Medication
       - Medication as indicated by the Clinical Practice Guidelines
       - Insulin syringes
    2. Counseling
       - Encourage diabetes patient to have contact with other diabetes patients in the village and peers
       - Behavioral changes regarding diet, exercise, smoking, alcohol
       - Risk factors for diabetic complications and means to prevent these
       - Adherence with and understanding of requirements for treatment
       - Check ability of self-monitoring, ask to see the patient self-management book, and discuss the records and progress towards targets

#### Required Documentation:

- Standard Patient Dossier Medical Records
- Hospital HEF Summary of Treatment Form
- Referral letter to higher-level hospital if necessary

Source: Guidelines for the Benefit Package and Provider Payment of the Health Equity Fund for the Poor

Table A. Profile of simulated population by HEF eligibility (N = 800,000)

| **Characteristic** | **HEF Eligibility** Poorest 20% (n = 160,000) | **HEF Eligibility** Poorest 30% (n = 240,000) |
| --- | --- | --- |
| **Age (at year 0)** |  |  |
| 26-30 years | 34,503 (22%) | 51,823 (22%) |
| 31-35 years | 28,334 (18%) | 42,479 (18%) |
| 36-40 years | 15,900 (10%) | 23,819 (10%) |
| 41-45 years | 20,009 (13%) | 29,896 (13%) |
| 46-50 years | 16,807 (11%) | 25,166 (10%) |
| 51-55 years | 17,406 (11%) | 26,377 (11%) |
| 56-60 years | 12,120 (8%) | 18,180 (8%) |
| 61-65 years | 9,674 (6%) | 14,428 (6%) |
| 66-70 years | 5,247 (3%) | 7,806 (3%) |
| **Sex** |  |  |
| Male | 76,809 (48%) | 115,204 (48%) |
| Female | 83,191 (52%) | 124,796 (52%) |
| **Initial state** |  |  |
| Healthy | 146,595 (92%) | 219,936 (92%) |
| Undiagnosed diabetes | 8,317 (5%) | 12,509 (5%) |
| Diet/lifestyle consultation   (no prescribed treatment) | 3,749 (2%) | 5,576 (2%) |
| Oral anti-diabetic therapy | 1,083 (1%) | 1,600 (1%) |
| Insulin therapy | 86 (0.1%) | 123 (0.1%) |
| Neuropathy | 117 (0.1%) | 172 (0.1%) |
| Retinopathy | 29 (0.02%) | 42 (0.02%) |
| Nephropathy | 24 (0.01%) | 41 (0.02%) |
| **Enrolled in HEF** | 120,431 (75%) | 180,312 (75%) |

Table B. Cases of catastrophic health expenditure by threshold (10%, 25%, 40% of household income)

| **Strategy** | **HEF Eligibility** | **OOP Coverage** | **Incremental CHE_10_** | **Incremental CHE_25_** | **Incremental CHE_40_** |
| --- | --- | --- | --- | --- | --- |
| Current standard | 20% | 100% | - | - | - |
|  |  | 80% | - | - | - |
|  | 30% | 100% | - | - | - |
|  |  | 80% | - | - | - |
| Diagnostics only | 20% | 100% | 664,700 | 545,380 | 480,480 |
|  |  | 80% | 202,720 | 197,760 | 193,620 |
|  | 30% | 100% | 799,860 | 610,580 | 532,820 |
|  |  | 80% | 202,680 | 197,360 | 193,740 |
| Drug therapy only | 20% | 100% | 589,040 | 501,680 | 444,800 |
|  |  | 80% | 139,120 | 151,520 | 162,400 |
|  | 30% | 100% | 775,360 | 601,440 | 528,000 |
|  |  | 80% | 230,080 | 244,640 | 245,600 |
| Complications only | 20% | 100% | 684,900 | 559,060 | 491,440 |
|  |  | 80% | 242,720 | 226,600 | 216,880 |
|  | 30% | 100% | 819,960 | 624,180 | 543,400 |
|  |  | 80% | 242,640 | 226,160 | 216,960 |
| Diagnostics +  Drug therapy | 20% | 100% | 618,100 | 512,340 | 454,320 |
|  |  | 80% | 66,540 | 101,120 | 117,140 |
|  | 30% | 100% | 753,040 | 577,500 | 506,540 |
|  |  | 80% | 68,040 | 102,280 | 118,460 |
| Drug therapy +   Complications | 20% | 100% | 642,920 | 530,160 | 467,640 |
|  |  | 80% | 124,340 | 144,620 | 151,780 |
|  | 30% | 100% | 778,380 | 595,660 | 520,640 |
|  |  | 80% | 124,800 | 144,480 | 152,320 |
| Diagnostics +   Drug therapy +   Complications | 20% | 100% | 665,200 | 544,380 | 479,260 |
|  |  | 80% | 144,800 | 156,940 | 161,580 |
|  | 30% | 100% | 800,960 | 610,540 | 532,860 |
|  |  | 80% | 145,120 | 157,040 | 163,120 |


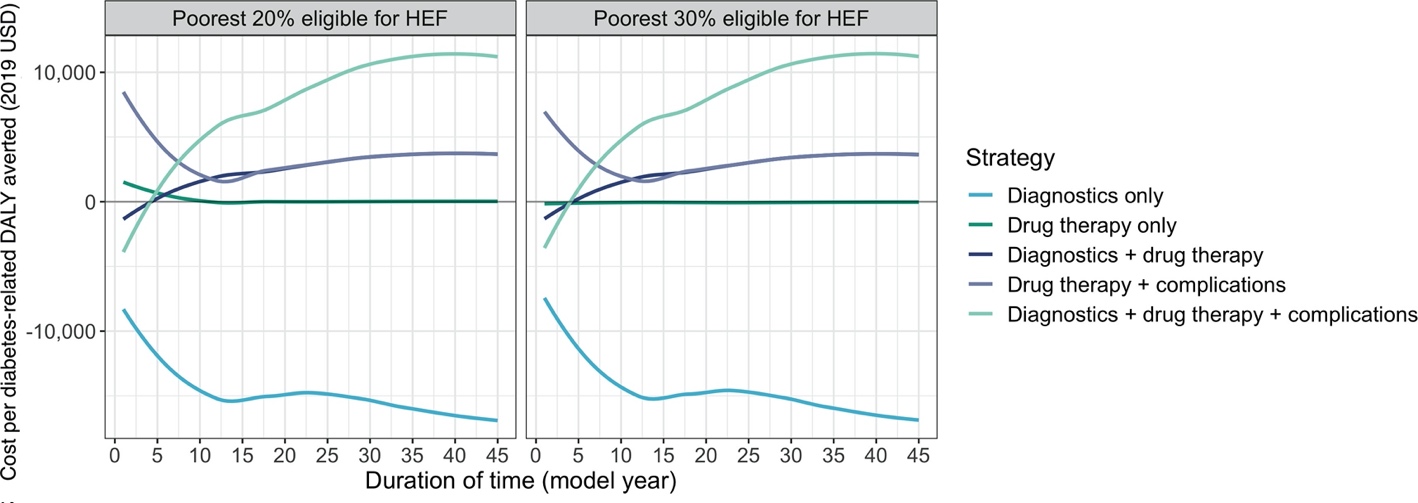


Figure A. Cost per diabetes-related DALY averted over time by strategy
Based on model design, coverage for treatment for diabetes-related complications had no additional impact on health. This strategy is not included in the figure.


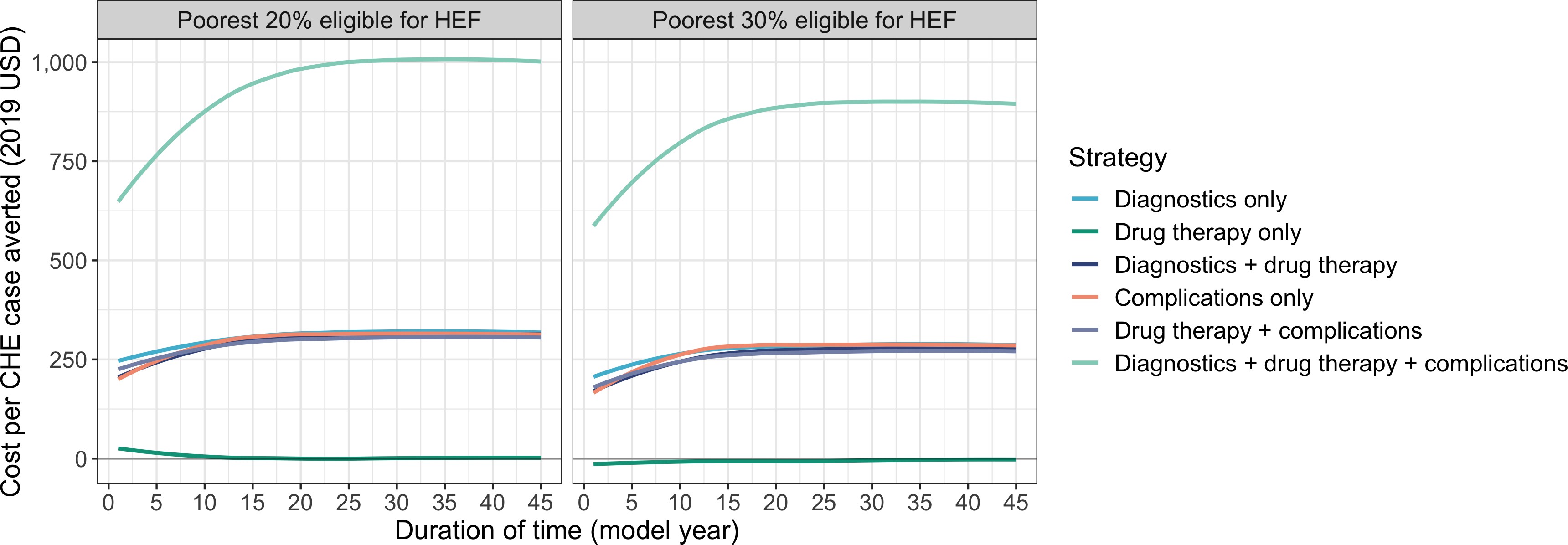


Figure B. Cost per diabetes-related case of CHE averted over time under 100% HEF coverage by strategy
