# Supplementary material for: Alleviating the burden of diabetes with Health Equity Funds: Economic evaluation of the health and financial risk protection benefits in Cambodia
Source: PLoS One. 2021 Nov 5;16(11):e0259628. doi: 10.1371/journal.pone.0259628 (PMC8570764; doi:10.1371/journal.pone.0259628)
Supplement: Attachment — Submitted filename: Response to Reviewers.docx [file pone.0259628.s002.docx]

**Response to reviewers’ comments:**

Reviewer #1: This is a highly sophisticated study which is not very easy to understand. However:

1. Prevention in high-risk individuals is totally ignored whereas it is critical in order to halt the progression of the diabetes "epidemic" and not overwhelm health services in LMICs. Considering also that there are several studies demonstrating the effectiveness of intensive lifestyle intervention studies in terms of curbing the incidence of diabetes, as well as their cost-effectiveness (see for instance Alouki K et al 2016). Was it not possible to integrate this in the model? If not, at least discuss this major limitation. Even if strengthening the Cambodia health system is on-going, it is doubtful that it could absorb the rising number of diabetes cases and provide quality management.

Thank you for this comment and highlighting the importance of prevention measures as some of the most cost-effective efforts to curb the incidence of diabetes. Indeed, we agree. This study focuses on what coverage can be provided by the Health Equity Funds in Cambodia to address the prevalence of diabetes. Unfortunately, prevention measures in the form of lifestyle or other daily activity interventions were determined to be outside of the scope of current HEF coverage policies, which largely focus on services that can be sought at health centers. Discussion of the importance of strategies aimed at preventing diabetes is included in the main text as the last paragraph of the section, “Financial risk protection and health systems strengthening for diabetes”.

2. Would the strategies of covering diagnostics only or of covering medication only not also allow for earlier diagnosis and treatment thereby contributing to curbing or delaying complications (Table 1)?

Thank you for this insightful comment. Yes, we agree that this is a possibility and a hoped-for impact of such a coverage strategy. Other models on diabetes have stratified cases by early diagnosis and late diagnosis of diabetes. We considered this approach as well. Unfortunately, for Cambodia, there is no information available describing how many diabetes cases are diagnosed early or later in disease progression, and there is limited data on the extent to which early vs. late diagnosis has an impact on treatment outcomes. As a result, we conservatively did not make this distinction in classification of diagnosis. Such an impact may drive more favorable results. Similarly, the typical timing of treatment in the diabetes trajectory remains unknown in Cambodia data sources. We have made revisions in the discussion to outline these limitations.

3. That coverage if diagnostics only would increase the negative health impact would have to be better substantiated.

The manuscript describes the negative health impact of the diagnostics only strategy due to disability weights attributed to daily psychological stress due to the knowledge of diagnosis result in negative health impact. These disability weights were used because they are standard as reported in the data source (IHME, 2017). In the scenario in which these disability weights are not acceptable for whatever reason, the diagnostics only strategy would have no impact. Following discussion of this point in the discussion section, the primary point made is that treatment subsequent to diagnosis would have truly positive health impacts.

4. The impact of user-fee removal on healthcare seeking has been assessed for conditions other than diabetes. References should be provided on this issue in the discussion, including for instance the study by Beaugé Y et al (2020).

Thank you for your comment and pointing us to this literature. Relevant discussion has been added to the introduction and discussion sections.

5. Please insert 'diabetes' in the title.

The title of the manuscript already includes the term ‘diabetes’ as follows: “Alleviating the burden of diabetes with Health Equity Funds: Economic evaluation of the health and financial risk protection benefits in Cambodia”.
